# Integration of population-level data sources into an individual-level clinical prediction model for dengue virus test positivity

**DOI:** 10.1126/sciadv.adj9786

**Published:** 2024-02-16

**Authors:** Robert J. Williams, Ben J. Brintz, Gabriel Ribeiro Dos Santos, Angkana T. Huang, Darunee Buddhari, Surachai Kaewhiran, Sopon Iamsirithaworn, Alan L. Rothman, Stephen Thomas, Aaron Farmer, Stefan Fernandez, Derek A. T. Cummings, Kathryn B. Anderson, Henrik Salje, Daniel T. Leung

**Affiliations:** ^1^ Division of Infectious Diseases, Department of Internal Medicine, University of Utah, Salt Lake City, UT, USA.; ^2^ Division of Epidemiology, Department of Internal Medicine, University of Utah, Salt Lake City, UT, USA.; ^3^ Department of Genetics, University of Cambridge, Cambridge, UK.; ^4^ Department of Virology, Armed Forces Research Institute of Medical Sciences, Bangkok, Thailand.; ^5^ Ministry of Public Health, Nonthaburi, Thailand.; ^6^ Institute for Immunology and Informatics and Department of Cell and Molecular Biology, University of Rhode Island, Providence, RI, USA.; ^7^ Department of Microbiology and Immunology, SUNY Upstate Medical University, Syracuse, NY, USA.; ^8^ Department of Biology, University of Florida, Gainesville, FL, USA.; ^9^ Emerging Pathogens Institute, University of Florida, Gainesville, FL, USA.; ^10^ Division of Microbiology and Immunology, Department of Pathology, University of Utah, Salt Lake City, UT, USA.

## Abstract

The differentiation of dengue virus (DENV) infection, a major cause of acute febrile illness in tropical regions, from other etiologies, may help prioritize laboratory testing and limit the inappropriate use of antibiotics. While traditional clinical prediction models focus on individual patient-level parameters, we hypothesize that for infectious diseases, population-level data sources may improve predictive ability. To create a clinical prediction model that integrates patient-extrinsic data for identifying DENV among febrile patients presenting to a hospital in Thailand, we fit random forest classifiers combining clinical data with climate and population-level epidemiologic data. In cross-validation, compared to a parsimonious model with the top clinical predictors, a model with the addition of climate data, reconstructed susceptibility estimates, force of infection estimates, and a recent case clustering metric significantly improved model performance.

## Teaser

Addition of population-level data to clinical data improved performance of a prediction model for dengue test positivity.

## INTRODUCTION

Acute febrile illness (AFI) is a common reason for seeking health care in low- and middle-income countries (LMICs) (*1*). Determination of AFI etiology is often limited by diagnostic testing capacity, given the wide spectrum of potential infectious agents. Inappropriate use of testing and treatment resources may result in poor outcomes, such as the high case fatality rates seen in admitted AFI patients (5 to 20%) (*2*–*7*). Dengue virus (DENV) is a major cause of AFI in LMICs, accounting for an estimated 390 million infections, 96 million illnesses, 2 million severe cases, and 21,000 deaths per year (*8*). The differentiation between dengue and other common causes of febrile illness is important to avoid misdiagnosis, which can lead to delays in initiation of effective treatment and inappropriate use of antibiotics (*9*). Because of the lack of pathognomonic clinical features that reliably distinguish dengue from other febrile illnesses, virological or serological laboratory confirmation is required for definitive diagnosis. While multiplexed tests that can quickly identify the causative pathogen are ideal, they are often unavailable in LMICs due to cost and insufficient laboratory infrastructure. Even rapid, point-of-care tests may be cost-prohibitive in LMICs (*10*). Accurate and cost-effective tools to better determine etiology of fever at the point of care are greatly needed to guide the use of diagnostics and therapeutics, conserving scarce health care resources.

Clinical decision support systems (CDSS) incorporating prediction models may offer a solution to better management of infectious diseases in low resource settings. CDSSs, such as applications on smartphone devices, can gather data from a range of online sources and implement sophisticated clinical prediction models that would be impractical for clinicians to calculate manually. CDSS have proven effective at improving therapeutic management and reducing unnecessary diagnostic tests in both high-income countries (*11*) and LMICs (*12*–*14*). In Bangladesh, an electronic CDSS was shown to improve clinical dehydration assessment and World Health Organization (WHO) diarrhea guideline adherence, as well as reduce nonindicated antibiotic use in children under five by 29% (*12*). Traditional predictive models generally incorporate clinical information that is obtained solely from the presenting patient. Predictive models that incorporate additional information—such as seasonal or climate predictors, location-specific historical prevalence, and characteristics of prior patients—have been shown to increase diagnostic accuracy and limit inappropriate antibiotic use (*14*–*16*).

The underlying probability of being infected by DENV varies by both space and time. The risk of DENV transmission depends on conditions that promote mosquito breeding, including when temperatures are warmer (*17*–*19*), and the risk of infection is influenced by local population immunity, as large outbreak years are typically followed by periods of low transmission (*20*–*22*). As most DENV transmission is highly focal, it means that population susceptibility profiles can be spatially heterogeneous at any time (*21*, *23*–*25*). Thus, our objective is to develop an improved clinical prediction model for dengue by integrating temporal and spatial (location-specific) parameters including climate data, clustering of recent cases, and population susceptibility estimates derived from seroprevalence or hospital data in the surrounding community. We demonstrate the potential for integrating location- and population-specific data sources into clinical prediction models. This approach has the potential to inform the development of improved tools to aid clinicians in diagnostic and therapeutic decision making for patients presenting with suspected dengue.

## RESULTS

Of the 12,833 participants in the clinical dataset, 5731 (45%) were confirmed to have DENV infection by polymerase chain reaction (PCR). DENV-positive patients were significantly younger (18 versus 22 years, *P* < 0.001; Table 1). Nearly all cases (97.8%) came from the 11 districts within Kamphaeng Phet province (Table 1). There was no significant difference between the probability of testing positive for males and females (*P* = 0.07); no other genders were reported. The probability of testing positive differed substantially by age, ranging from 26% for those <4 years to 58% for those 15 to 19 years of age (Table 2). Patients between the ages of 10 and 14 years, 15 and 19 years, and 5 and 9 years comprised the largest proportion of cases (23, 18, and 16%, respectively), while older patients comprised a much smaller proportion of cases (30 to 34 years, 5%; 35 to 39 years, 4%).

**Table 1.** Age, gender, and top discriminative symptoms by DENV positivity. Locations listed are the 11 provinces in Kamphaeng Phet.

	Overall (*N* = 12,833)*	DENV negative (*N* = 7,102)*	DENV positive (*N* = 5,731)*	*P* value^†^
Age (mean, SD)	21 (15)	22 (18)	18 (11)	<0.001
Female	6,401 (50)	3,491 (49)	2,910 (51)	0.068
Symptoms				
Cough	4,741 (37)	3,057 (43)	1,684 (29)	<0.001
Nausea	6,227 (49)	3,051 (43)	3,176 (55)	<0.001
Fever	11,467 (89)	6,129 (86)	5,338 (93)	<0.001
Headache	9,146 (71)	4,797 (68)	4,349 (76)	<0.001
Rhinitis	2,165 (17)	1,455 (20)	710 (12)	<0.001
Pharyngitis	3,534 (28)	2,113 (30)	1,421 (25)	<0.001
Location				
District				<0.001
Bueng Samakkhi	226 (1.8)	166 (2.3)	60 (1.0)	
Khanu Woralaksaburi	910 (7.1)	522 (7.4)	388 (6.8)	
Khlong Khlung	733 (5.7)	397 (5.6)	336 (5.9)	
Khlong Lan	945 (7.4)	645 (9.1)	300 (5.2)	
Kosamphi Nakhon	750 (5.8)	407 (5.7)	343 (6.0)	
Lan Krabue	556 (4.3)	333 (4.7)	223 (3.9)	
Mueang Kamphaeng Phet	5,780 (45)	2,910 (41)	2,870 (50)	
Pang Sila Thong	571 (4.4)	324 (4.6)	247 (4.3)	
Phran Kratai	1,186 (9.2)	684 (9.6)	502 (8.8)	
Sai Ngam	609 (4.7)	363 (5.1)	246 (4.3)	
Sai Thong Watthana	288 (2.2)	178 (2.5)	110 (1.9)	
Province				
Kamphaeng Phet	12,554 (97.8)	6,929 (97.5)	5,625 (98.2)	

*Mean (SD); *n* (%).

†Wilcoxon rank sum test; Pearson’s chi-square test.

**Table 2.** The AUCs and CIs by base model, compared to base model plus inclusion of additional data sources. “Clinical” indicates the inclusion of the top three clinical predictors, “Climate” indicates the inclusion of climate predictors, “RS” indicates the inclusion of reconstructed susceptibility estimates derived using national surveillance data, “FOI” indicates the inclusion of force of infection estimates derived using cohort data, and “Cluster” indicates the recent case cluster metric.

Model	AUC (%)	95% CI
Clinical*Climate*RS*FoI*Cluster	70.0	67.9–71.9
Clinical*Climate*RS*Cluster	69.5	67.5–71.5
Clinical*Climate*FoI*Cluster	69.2	67.2–71.2
Clinical*Climate*Cluster	68.8	66.8–70.8
Clinical*Climate*RS*FoI	68.7	66.7–70.7
Clinical*Cluster	68.7	66.7–70.7
Clinical*FoI*Cluster	68.5	66.5–70.6
Clinical*Climate*RS	68.4	66.4–70.5
Clinical*RS*FoI*Cluster	68.4	66.4–70.4
Clinical*RS*Cluster	68.2	66.1–70.2
Clinical*Climate*FoI	68.1	66.1–70.1
Clinical*FoI	67.7	65.7–69.8
Clinical*RS*FoI	67.6	65.5–69.6
Climate*RS*FoI*Cluster	67.5	65.5–69.6
Clinical*RS	67.5	65.4–69.5
Clinical*Climate	67.2	65.2–69.3
Clinical	67.0	65–69.1
Climate*RS*Cluster	66.8	64.8–68.9
Climate*RS	65.7	63.6–67.8
RS*Cluster	65.7	63.6–67.7
RS	65.6	63.5–67.7
Climate*FoI*Cluster	64.7	62.6–66.8
Climate*Cluster	60.5	58.3–62.7
Climate	58.7	56.5–60.9
Cluster	56.4	54.2–58.6
FoI	57.0	54.8–59.2

We found that there were significant differences in many of the clinical symptoms reported by DENV-positive and DENV-negative patients. Table 1 lists the top discriminative symptoms between the groups based on random forest and logistic regression. The most common symptom reported was fever, followed by headache. In univariate analysis, we found that individuals with fever, chills, malaise, retro-orbital pain, nausea, headache, and vomiting were significantly more likely to test positive for DENV, and individuals with cough, rhinitis, and pharyngitis were significantly less likely to test positive for DENV (table S2).

**Fig. 1.**
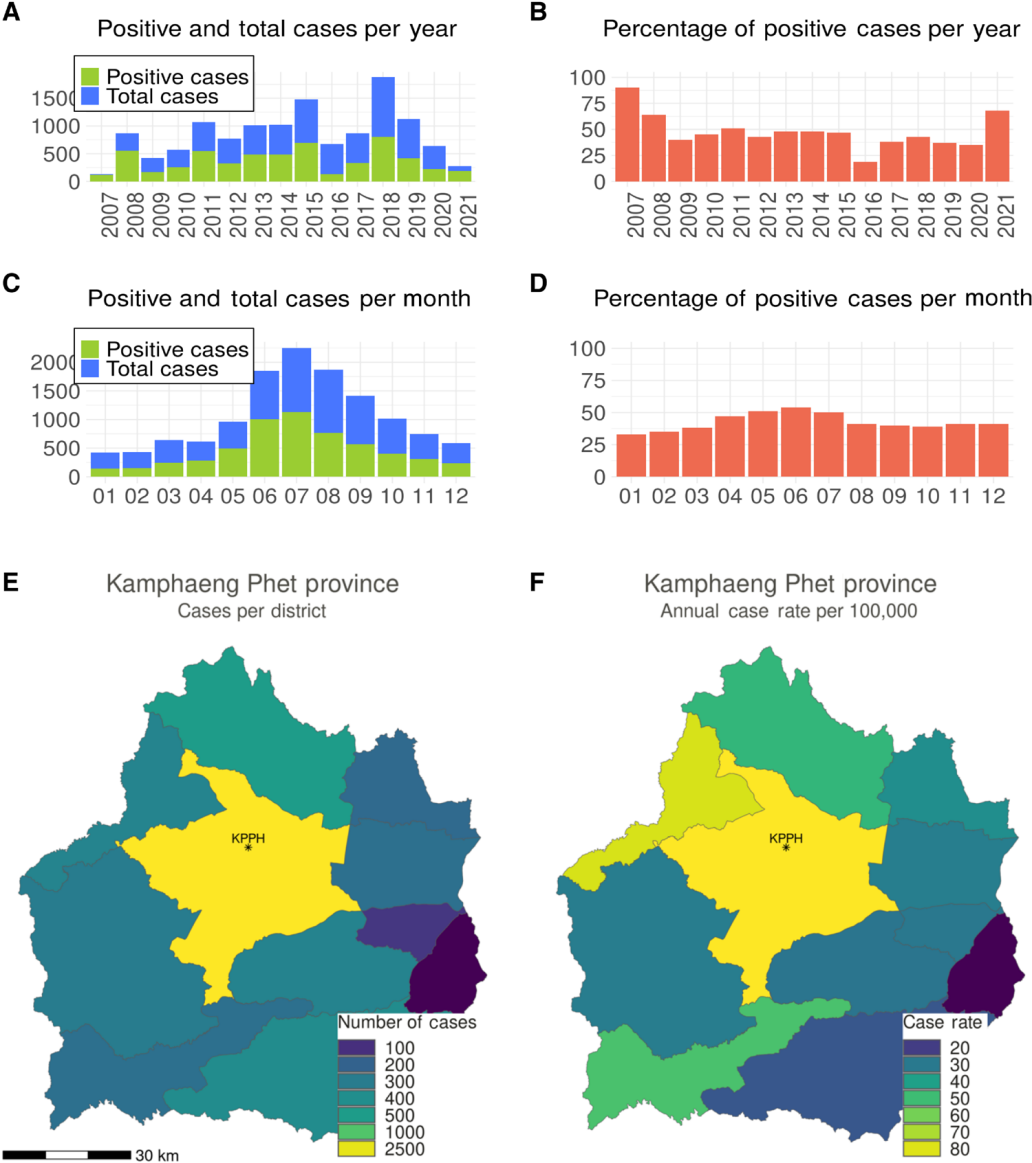
DENV cases at KPPH, Thailand, 2007–2021. The number of DENV cases (green) over total cases (blue) as proportion of AFI cases by year (**A**) and month (**C**) and the percentage of positive cases by year (**B**) and month (**D**) over the study period. A map of Kamphaeng Phet Province and its 11 districts. Colors indicate the number of positive cases (**E**) and the annual case rate per 100,000 persons (**F**) within each district between 2007 and 2021.

When we examined the proportion of positive cases to total cases by year and month, we found that both total and positive cases significantly increased in the months between June and September (*P* < 0.001, χ^2^ test). The proportion of positive cases differed substantially by year (*P* < 0.001, χ^2^ test), ranging from 19% in 2016 to 90% in 2017. The period of lowest test positivity in 2016 and 2017 coincided with the Zika virus epidemic in the country (Fig. 1).

### Model performance evaluation using only clinical predictors and parsimonious variable selection

**Fig. 2.**
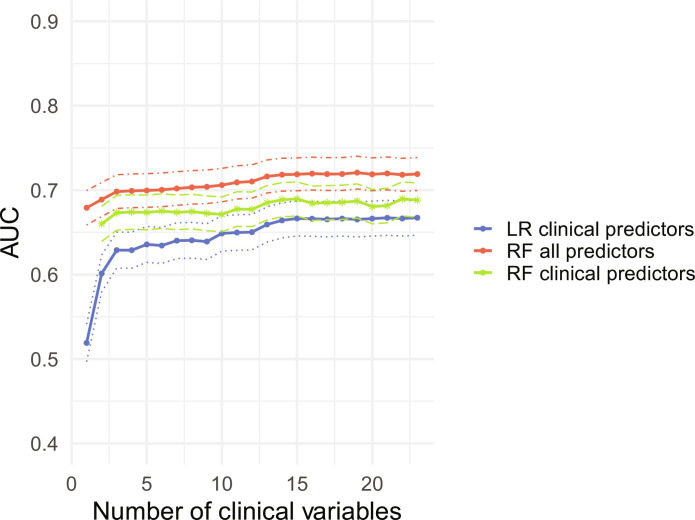
Average AUC and 95% CIs from cross-validation (100 iterations) for random forest and logistic regression models. The red line indicates an random forest (RF) model with all other predictors (climate, reconstructed susceptibilities estimates, FoI estimates, and prior patients) included. The green line indicates an RF model that includes only clinical predictors. The blue line indicates an logistic regression (LR) model with only clinical predictors included. The dotted lines indicate CIs.

We first assessed the performance of the model using a traditional clinical prediction model, which only includes the presenting patient’s information. A random forest classifier using all 23 clinical features resulted in an average area under the receiver operator characteristic curve (AUC) of 69.5% [95% confidence interval (CI): 67.5 to 71.5] from repeated cross-validation. To determine the optimal number of variables for a parsimonious prediction model, we used a random forest classifier to analyze the improvement in model performance with each additional clinical variable included. Figure 2 shows the improvement in AUC with each additional variable using two random forest classifiers—one with all other predictors and the other using only clinical data—as well as a logistic regression model using only clinical variables. Performance leveled off with three clinical variables: age, cough, and nausea. Using a model with only these three predictors, we achieve an average AUC of 67.0% (95% CI: 65.0 to 69.1). Table S3 shows the relative frequency of these variables by age group. We demonstrate the direction and magnitude of the effect of the top predictors by generating partial dependence plots from random forest and logistic regression classifiers (fig. S1).

### Addition of climate data to the clinical parameter model resulted in an improved AUC

**Fig. 3.**
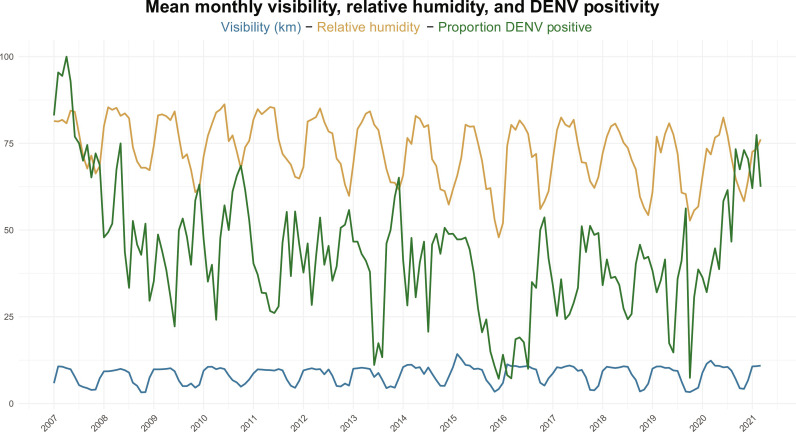
The monthly relative humidity (orange) and visibility (blue) in Thailand over the study period, compared with rates of DENV (green). For each case, we gathered the nearest NOAA weather station’s climate data, lagged by three months, and averaged those data for each month.

Next, we fit models using climate data. To appropriately adjust lag time for each climate variable, we fit a random forest classifier using only climate variables and assessed the variables of importance by AUC. A random forest model with recent and lagged aggregated climate data without clinical predictors resulted in an AUC of 58.7% (95% CI: 56.5 to 60.9). We found that the best performing climate variables were visibility, relative humidity, wind speed, and precipitation, all lagged by 3 months. We examined the relationship between the top two performing climate predictors—visibility and relative humidity—with the proportion of positive cases each month (Fig. 3). For each climate predictor, table S4 lists the odds ratio and compares the mean of each predictor by DENV-positive or DENV-negative groups. When combined with the top three clinical variables, climate data performed similarly (AUC of 67.2%, 95% CI: 65.2 to 69.3) as clinical data alone (AUC of 67.0%, 95% CI: 65.0 to 69.1) (median *P* = 0.60, 2% *P* < 0.05). However, when climate data were combined with all other predictors, model performance improved from an AUC of 68.4% (95% CI: 66.4 to 70.4) to an AUC of 70.0% (95% CI: 67.9 to 71.0; median *P* = 0.07, 45% *P* < 0.05). To assess whether integrating more location-specific climate data would improve performance, we fit models using climate data from each case’s home district; however, model performance did not noticeably change. Table 2 shows the AUCs for the clinical base model, compared to the base model plus the inclusion of additional data sources.

### Addition of RS estimates to the clinical parameter model resulted in an improved AUC

Using historical hospital case data from the province, we obtained estimates of the size of the susceptible population by age for each year (across all subdistricts in the province). In our predictive model, we used the prior year’s reconstructed susceptibility (RS) estimates. Using logistic regression, we found that secondary RS estimates performed better than primary RS estimates [60.7% (95% CI: 58.6 to 62.9) versus 52.3% (95% CI: 50.1 to 54.6)]. When added to a random forest classifier with climate and/or clinical predictors, the inclusion of RS estimates consistently resulted in higher AUCs (Table 2). When added to the top three clinical parameters alone, RS estimates nonsignificantly improved model performance from an AUC of 67.0% (95% CI: 65.0 to 68.8) to an AUC of 67.5% (95% CI: 65.4 to 69.5) (median *P* = 0.40, 9% *P* < 0.05). Last, a model including all predictors resulted in higher AUCs than a model without RS (median *P* = 0.09, 32% *P* < 0.05).

### Addition of subdistrict-specific FoI estimates to the clinical parameter model resulted in an improved AUC

We incorporated force of infection (FoI) estimates for each age by subdistrict using data from a local cohort study. This assumes that the underlying differences in the FoI are constant in time. Using logistic regression, FoI estimates had an AUC of 57.0% (95% CI: 54.8 to 59.2). The inclusion of FoI estimates leads to increases in AUC when added to the top clinical predictors, when added to clinical predictors and climate data, and when added to clinical predictors, climate predictors, and RS estimates (Table 2). When included with all other predictors, a model with FoI estimates nonsignificantly improved performance compared to a model without FoI estimates (median *P* = 0.30, 23% *P* < 0.05).

### Addition of the case clustering metric to the clinical parameter model resulted in an improved AUC

Last, we fit a model that assessed for clustering of recent cases based on prior patients presenting to the Kamphaeng Phet Provincial Hospital (KPPH). Using logistic regression, we found that the case clustering metric (the number of positive cases in the subdistrict over the past 30 days divided by the total number of cases from that subdistrict in the study period) had an AUC of 56.4% (95% CI: 54.2 to 58.6). We found that the use of the case clustering metric consistently improved model performance. Stratifying by the finer spatial size of subdistrict consistently outperformed models with prior patients stratified by province. When added to the top performing clinical variables, model performance significantly improved (median *P* = 0.02, 60% of *P* < 0.05). When compared to a model with all predictors except cluster of recent cases, the inclusion of this predictor significantly improved model performance (median *P* = 0.007, 79% *P* < 0.05).

Last, when comparing a model including all predictors with a model including only the top clinical predictors, model performance improved from an AUC of 67.0% (95% CI: 65.0 to 69.1) to an AUC of 70.0% (95% CI: 67.9 to 71.9) (median *P* = 0.006, 87% *P* < 0.05). Our model had a sensitivity of 55.3%, a specificity of 70.2%, a positive predictive value (PPV) of 60.0%, and a negative predictive value (NPV) of 66.1%.

## DISCUSSION

Insufficient diagnostic testing capacity in LMICs necessitates innovative approaches to support clinical decision-making. Here, we present a predictive model for DENV infection that integrates multiple sources of information both intrinsic and extrinsic to the patient, including climate data, clinical data, seroprevalence-based susceptibility estimates, and historical information from prior patients, which results in improved predictive performance. While the model with all predictors included did significantly outperform the base parsimonious model with only clinical predictors (median *P* = 0.006, 87% *P* < 0.05), whether the additional 3.0% improvement in AUC is clinically useful may be case and clinician dependent. Certain components of our model require data from sero-surveillance, which may not be accessible in all communities. However, simplifying the model by including only the top clinical predictors and the case cluster metric alone results in an AUC decrease of only 1.3%. These metrics are more readily obtainable and, notably, do not necessitate laboratory resources. Nevertheless, we believe that the results demonstrate a proof of concept that seroprevalence-based susceptibility estimates and climate data can be used to improve predictive performance and may be useful to augment prediction in other communicable diseases.

There is a lack of information on the deficiency of testing capacity both in Thailand and globally in LMICs. Accurately quantifying the true extent of diagnostic testing deficiencies is challenging as LMICs often lack robust national surveillance systems. In a Brazilian study between the years 2010 and 2019, where every suspected case of dengue was recorded in a national surveillance database, only 11% of the 350,000 cases of suspected dengue infection were ultimately tested (*26*). If we extrapolate the results from Brazil to other LMICs, as much as 90% of dengue-like illness may go undiagnosed, highlighting the need for tools to bridge the diagnostic gap.

In contrast to most dengue diagnostic models, which rely in part on laboratory data, our model relies solely on clinical indicators, making it accessible to clinicians without laboratory resources. For reference, when compared to a multiple regression model from Honduras that used only clinical predictors, our model had a lower sensitivity (55% versus 86%) and PPV (60% versus 75%) and a higher specificity (70% versus 27%) and NPV (66% versus 44%) (*27*). Models that integrate laboratory values, such as complete blood count and hepatic function tests, tend to perform better than models using only clinical predictors, such as a Bayesian network model from Thailand (sensitivity, 74%; specificity, 79%; PPV, 75%; NPV 79%) (*28*), a multiple regression model from Sri Lanka (sensitivity, 49%; specificity, 85%; PPV, 70%; NPV, 70%) (*29*), and a multiple regression model from Brazil (sensitivity, 80%; specificity, 71%) (*30*).

DENV transmission can exhibit temporal and geographical heterogeneity even at fine spatial scales, with variations observed even among neighboring villages (*31*–*33*). We thus used patient-extrinsic (location-specific) data sources in our models. The improvement in AUC with finer spatial units suggests that population-level spatial heterogeneity exists at the district level and can be applied to individual-level clinical prediction. We expect further improvements in predictive performance if finer-scale location became routinely available for case data, such as to the community level. The improvement with the use of either the province or district level case clustering metric highlights the utility of temporal predictors in clinical prediction DENV models.

Spatial heterogeneity in dengue incidence may be explained in part by micro-climates, which can modify transmission dynamics at small scales. For example, within urban heat islands, temperature variations of up 10°C compared to other city areas may create conditions more conducive to dengue transmission in cooler temperatures (*34*). We collected all climate data from the provincial weather station in Kamphaeng Phet. We attempted to integrate climate data at a more localized level; however, several subdistricts do not have weather stations or weather station data were incomplete. When fitting models using data from all districts in Kamphaeng Phet, however, we found similar results.

Transmission of DENV occurs in a seasonal pattern, and several climate variables have been found to increase DENV transmission and/or vector populations (*17*–*19*, *35*, *36*). While prior studies have demonstrated associations between climate variables like average precipitation, relative humidity, temperature, and wind speed, with varying lag times between 0 and 3 months, and dengue incidence (*37*–*40*), our predictive-based analytic framework is not intended to examine causal or associative relationships between climate variables and the outcome of dengue incidence. Our findings suggest that site-specific climate variables aid in site-specific models to predict DENV infection. While visibility has not been found to be associated with dengue incidence, we found that it was the most important climate predictor. It is plausible that visibility serves as a proxy indicator for an underlying factor that affects dengue incidence, such as air pollution, which has been postulated as a contributing factor (*41*, *42*). Appropriate lag times would need to be tuned to different sites. For use in a clinical decision support tool, the most recent climate variables could be gathered from online weather sources based on smartphone-based detection of GPS location. An optimal utilization of this model would be through a smartphone application, as there is a scarcity of electronic medical record availability in LMICs. This would necessitate access to a smart phone device and internet connection; however, clinicians and frontline health care workers increasingly have access to smartphone devices, even in remote areas of LMICs (*43*).

There were significant differences between DENV-positive and DENV-negative patients in 16 of the 22 clinical symptoms collected on presentation, consistent with features known to distinguish dengue from other illnesses (*44*, *45*). To minimize clinician input requirements (*46*), we used random forest regression to identify the optimal variables to derive a parsimonious model. We were able to achieve near-optimal performance with only three clinical variables—age, nausea, and cough. Numerous multivariable models based on clinical presentation have been developed to identify dengue infection in patients with AFI. In a review of published logistic regression prediction models, rash and/or petechiae was the most frequently identified predictor (four of seven models) to discriminate between DENV-positive and DENV-negative patients. When evaluated, the absence of cough was found to be a predictor in 33% of models. Nausea, which was evaluated in four logistic regression models, did not achieve significance in any model. Our results differ from those found in many logistic regression models and align with more intricate models for DENV diagnosis. Models using deep neural networks (*47*), random forest (*48*), and gradient boosting (XGBoost) (*49*) noted that age was the best clinical discriminative predictor. These models did not include cough or nausea as variables for assessment. We found that with the input of as little as one clinical variable—age—along with other predictors can provide useful clinical information (AUC: 67.9%, 95% CI: 65.6 to 70.0), especially in cases where other symptoms cannot be easily obtained, such as in nonverbal or comatose patients.

We show that RS estimates, which reflect the transmission dynamics of disease and the susceptible proportion of a population, improve individual-level clinical prediction on their own. However, there are several factors that make the use of RS estimates problematic, and we favor the use of other location-specific predictors. First, RS estimates may be more difficult to obtain across different settings. Moreover, RS estimates may not serve as a reliable indicator of protection against DENV, as they represent a mixed concept—immunity may reflect protection due to herd immunity or may indicate increased risk of dengue infection, as higher levels of immunity may reflect higher viral circulation of the multiple DENV serotypes with substantial immunologic cross-reactivity. Last, RS estimates are themselves derived from a model and so should be considered with caution.

Our study has several limitations. First, our model was constructed using data from a single center and testing was limited to patients suspected of having dengue infection, potentially hindering the model’s generalizability to a broader population. Similarly, as there was inherent heuristic bias in the patients selected for testing, the clinical components of the model reflect this specific population, meaning that other important predictors of dengue infection, such as fever, were already included in the clinician’s decision-making. Our results were limited to internal cross-validation; further studies for external validation are necessary. Last, our assessment of the use of spatial dynamics in DENV transmission was limited as cases were only matched to each district rather than subdistrict or village. In the future, models that integrate cases based on a finer spatial scale may better assess the role of a patient’s residing location in prediction. Despite these limitations, we demonstrate that predictive models that include patient-extrinsic location-specific elements can improve prediction and allow for parsimonious models that minimize clinician input and should be considered in future work on clinical prediction and decision support tools.

## METHODS

### Location

Kamphaeng Phet is a province in north-central Thailand, which is located 350 km north of Bangkok and has a population of 725,000 people in a mostly rural and semirural setting (*33*, *50*). We used data collected from patients presenting to KPPH, a large, tertiary care hospital in the province to identify clinical predictors that could discriminate between DENV-infected and uninfected patients (*33*, *50*).

### Hospital-based suspected dengue patient data

We used data on over 12,000 patients presenting to KPPH with suspected dengue between August 2007 and December 2021. The data were collected by the U.S. Army Medical Directorate–Armed Forces Research Institute of Medical Sciences. As DENV testing in this hospital is provided free of charge and this is a highly DENV-endemic region, individuals will be tested for DENV infection if there is any suspicion of dengue, however minor. This provides an excellent test case to understand whether individual or location-specific risk factors are associated with testing positive for DENV.

For all suspected dengue cases, we used demographic and clinical information including patient age, sex, home village, admission diagnosis, date of admission, presenting symptoms, and DENV PCR status. The following signs and symptom were recorded as binary variables: fever, chills, malaise, rhinitis, rash, sore throat, seizure, cough, nuchal rigidity, eye pain, nausea, headaches, vomiting, joint pain, abnormal movements, anorexia, myalgias, diarrhea, dark urine, abdominal pain, and bleeding. DENV infection was evaluated using reverse transcription PCR. We recorded the residence of each patient to the district (Amphoe) level using detailed base maps of the region.

### Climate variables using NOAA data

Climate and seasonal factors such as temperature, precipitation, and humidity influence vector populations and DENV transmission (*17*–*19*, *35*). We used the R package GSODR to gather climate data from the central most National Oceanic and Atmospheric Administration (NOAA) weather station in the province of Kamphaeng Phet, Thailand, which included mean daily temperature, precipitation, dew point, relative humidity, sea level pressure, visibility, and wind speed. To better reflect seasonal trends, we aggregated data in 14-day increments before the day of the DENV infection prediction. As climate can alter vector feeding behavior (*19*, *36*), we used aggregated climate predictors in the 2 weeks before case presentation. In addition, climate in the months before outbreaks can influence both vector population dynamics and viral replication (*19*, *35*). To determine the appropriate lag time for each climate variable, we constructed a random forest classifier with climate variables lagged at 1, 2, and 3 months. Using the R package “vip,” we calculated each variable of importance by AUC and used the best performing lag time for each climate variable.

### Estimates of temporal changes in population susceptibility using national surveillance system data

We estimate population susceptibility data using age-specific case data from the national surveillance system using data from Kamphaeng Phet province only. We note that most of the cases in this dataset are suspected DENV cases (i.e., without confirmatory testing). We have previously developed models to explicitly link underlying infection risks to the observed age distribution of cases by age and year to estimate annual age-specific FoI in provinces of Thailand up until 2017 (*51*). The estimates can be used to reconstruct the buildup of immunity in populations by age. Here, we reconstruct population susceptibilities in Kamphaeng Phet going into each year, using only data before the year, to mimic the real-world use, where only prior years’ data are available. As dengue disease severity is greatest for secondary infections, we consider two alternative formulations to define susceptibility to disease. First, we consider complete susceptibility, where we use the estimates of the proportion of individuals of an age group and year that are completely seronaive. Second, we consider the proportion of individuals of an age group and year that have experience one prior infection and are therefore at risk of increased risk of severe disease.

### Estimates of spatial differences in the underlying FoI using seroprevalence data from a cohort study

To estimate underlying spatial differences in the FoI in the province, we make use of a DENV cohort study in the region, where healthy individuals of all ages from throughout Kamphaeng Phet province have provided blood (*52*). The cohort is ongoing. We use data from samples collected during baseline blood draws that occurred between 2015 and 2021. Hemagglutination inhibition assays were used to characterize immunity to the four DENV serotypes; individuals were considered seropositive if they had a titer of 10 or greater to any serotype. We have previously used these seroprevalence data to estimate the underlying mean FoI and the proportion of the population that are susceptible to DENV infection in different subdistricts in the province (*53*). Here, we use these subdistrict-specific estimates to characterize underlying heterogeneity in the FoI in the province. As the cohort data come from 2015 to 2021, however, much of the hospital case data we are working with come from before the cohort, we are assuming that the FoI is stable in time within any location.

### Spatial clustering of positive cases based on prior patients presenting to the hospital

The local clustering of positive cases from a single area may signal local ongoing transmission. To assess for a temporal and spatial relationship between cases, we stratified cases that presented to KPPH by both district and province and then summed the number of positive cases in the 30 days before presentation divided by the total cases over the study period from that area.

### Statistical analysis and modeling

We fit random forest classifiers to predict DENV infection. Random forests are a machine learning method that constructs a multitude of decision trees and averages over them to obtain a prediction robust to nonlinearities and interactions between covariates and has been widely applied to biomedical sciences for both classification and regression (*54*, *55*).

We initially identified the subset of clinical symptoms that were most informative of true infection status. To do this, we fit random forest models using only clinical predictors and then used the R package vip to calculate the variable of importance by AUC for each clinical variable. We determined a variable’s importance by calculating the change in AUC after permuting, or randomly shuffling each predictor. To attempt to achieve the most parsimonious prediction rule (i.e., the best predictive model requiring the fewest variables to be input by clinicians), we fit random forest and logistic regression models using training data with consecutively increasing clinical predictor set sizes based on the order of importance and applied this to the test set to determine the smallest model with the best performance. Next, we incorporated the patient-extrinsic factors. We fit each random forest classifier using 1000 decision trees and used the default number of variables to be randomly considered at each node split (*mtry* = square root of number of candidate variables). In the construction of our predictive models, we input climate predictors, age, susceptibility estimates, and the case clustering metric as continuous variables, and we input the optimized clinical predictors as binary presence or absence categorical variables. Missing predictor data were imputed using the R package “RandomForest.”

We used logistic regression for each predictor to create a univariate comparison between DENV-positive and DENV-negative cases. We fit multiple logistic regression models to compare the performance of parsimonious models with a random forest classifier using the same number of predictors.

To assess predictive performance for both random forest and logistic regression models, we used repeated cross-validation using 80% training/20% testing splits with 100 iterations. No testing data were used when training the model. In each iteration, predictions on the test set were produced and corresponding measures of performance were obtained. To determine overall model performance, we averaged the AUC and CIs for the 100 iterations. To determine statistical significance between models, we used a bootstrap method over 100 iterations, which involves resampling the data with replacement multiple times, creating bootstrap samples. For each bootstrap sample, receiver operating characteristic (ROC) curves were generated and the differences between the curves were computed. All analyses were completed using R version 4.2.0, and model development/validation was completed in accordance with the TRIPOD checklist (table S1).

### Ethical considerations

This study was approved by the institutional review boards of the Thai Ministry of Public Health and Walter Reed Army Institute of Research (no. 2119) and the University of Utah (IRB_00150106).

## References

[1] J. Osborn, T. Roberts, E. Guillen, O. Bernal, P. Roddy, S. Ongarello, A. Sprecher, A.-L. Page, I. Ribeiro, E. Piriou, A. Tamrat, R. de la Tour, V. B. Rao, L. Flevaud, T. Jensen, L. McIver, C. Kelly, S. Dittrich, Prioritising pathogens for the management of severe febrile patients to improve clinical care in low- and middle-income countries. BMC Infect. Dis. 20, 117 (2020). 32041536 10.1186/s12879-020-4834-1 PMC7011354

[2] N. Prasad, D. R. Murdoch, H. Reyburn, J. A. Crump, Etiology of severe febrile illness in low- and middle-income countries: A systematic review. PLOS ONE 10, –e0127962 (2015). 10.1371/journal.pone.0127962 PMC4488327 26126200

[3] D. R. Feikin, B. Olack, G. M. Bigogo, A. Audi, L. Cosmas, B. Aura, H. Burke, M. K. Njenga, J. Williamson, R. F. Breiman, The burden of common infectious disease syndromes at the clinic and household level from population-based surveillance in rural and urban Kenya. PLOS ONE 6, e16085 (2011). 21267459 10.1371/journal.pone.0016085 PMC3022725

[4] L. K. Archibald, M. O. den Dulk, K. J. Pallangyo, L. B. Reller, Fatal Mycobacterium tuberculosis bloodstream infections in febrile hospitalized adults in Dar es Salaam, Tanzania. Clin. Infect. Dis. 26, 290–296 (1998). 9502444 10.1086/516297

[5] K. Chheng, M. J. Carter, K. Emary, N. Chanpheaktra, C. E. Moore, N. Stoesser, H. Putchhat, S. Sona, S. Reaksmey, P. Kitsutani, B. Sar, H. R. van Doorn, N. H. Uyen, L. Van Tan, D. Paris, S. D. Blacksell, P. Amornchai, V. Wuthiekanun, C. M. Parry, N. P. J. Day, V. Kumar, A prospective study of the causes of febrile illness requiring hospitalization in children in Cambodia. PLOS ONE 8, e60634 (2013). 23593267 10.1371/journal.pone.0060634 PMC3621876

[6] J. A. Crump, A. B. Morrissey, W. L. Nicholson, R. F. Massung, R. A. Stoddard, R. L. Galloway, E. E. Ooi, V. P. Maro, W. Saganda, G. D. Kinabo, C. Muiruri, J. A. Bartlett, Etiology of severe non-malaria febrile illness in Northern Tanzania: A prospective cohort study. PLOS Negl. Trop. Dis. 7, e2324 (2013). 23875053 10.1371/journal.pntd.0002324 PMC3715424

[7] F. N. Ssali, M. R. Kamya, F. Wabwire-Mangen, S. Kasasa, M. Joloba, D. Williams, R. D. Mugerwa, J. J. Ellner, J. L. Johnson, A prospective study of community-acquired bloodstream infections among febrile adults admitted to Mulago Hospital in Kampala, Uganda. J. Acquir. Immune Defic. Syndr. Hum. Retrovirol. 19, 484–489 (1998). 9859962 10.1097/00042560-199812150-00007

[8] S. Bhatt, P. W. Gething, O. J. Brady, J. P. Messina, A. W. Farlow, C. L. Moyes, J. M. Drake, J. S. Brownstein, A. G. Hoen, O. Sankoh, M. F. Myers, D. B. George, T. Jaenisch, G. R. W. Wint, C. P. Simmons, T. W. Scott, J. J. Farrar, S. I. Hay, The global distribution and burden of dengue. Nature 496, 504–507 (2013). 23563266 10.1038/nature12060 PMC3651993

[9] J. A. Crump, S. Gove, C. M. Parry, Management of adolescents and adults with febrile illness in resource limited areas. BMJ 343, d4847 (2011). 21824901 10.1136/bmj.d4847 PMC3164889

[10] P. Yager, G. J. Domingo, J. Gerdes, Point-of-care diagnostics for global health. Annu. Rev. Biomed. Eng. 10, 107–144 (2008). 18358075 10.1146/annurev.bioeng.10.061807.160524

[11] T. J. Bright, A. Wong, R. Dhurjati, E. Bristow, L. Bastian, R. R. Coeytaux, G. Samsa, V. Hasselblad, J. W. Williams, M. D. Musty, L. Wing, A. S. Kendrick, G. D. Sanders, D. Lobach, Effect of clinical decision-support systems. Ann. Intern. Med. 157, 29–43 (2012). 22751758 10.7326/0003-4819-157-1-201207030-00450

[12] S. Bilal, E. Nelson, L. Meisner, M. Alam, S. Al Amin, Y. Ashenafi, S. Teegala, A. F. Khan, N. Alam, A. Levine, Evaluation of standard and mobile health-supported clinical diagnostic tools for assessing dehydration in patients with diarrhea in rural bangladesh. Am. J. Trop. Med. Hyg. 99, 171–179 (2018). 29761756 10.4269/ajtmh.17-0648 PMC6085783

[13] F. F. Tuon, J. Gasparetto, L. C. Wollmann, T. P. Moraes, Mobile health application to assist doctors in antibiotic prescription - An approach for antibiotic stewardship. Braz. J. Infect. Dis. 21, 660–664 (2017). 28941393 10.1016/j.bjid.2017.08.002 PMC9425452

[14] S. C. Garbern, E. J. Nelson, S. Nasrin, A. M. Keita, B. J. Brintz, M. Gainey, H. Badji, D. Nasrin, J. Howard, M. Taniuchi, J. A. Platts-Mills, K. L. Kotloff, R. Haque, A. C. Levine, S. O. Sow, N. H. Alam, D. T. Leung, External validation of a mobile clinical decision support system for diarrhea etiology prediction in children: A multicenter study in Bangladesh and Mali. eLife 11, e72294 (2022). 35137684 10.7554/eLife.72294 PMC8903833

[15] A. M. Fine, J. S. Brownstein, L. E. Nigrovic, A. A. Kimia, K. L. Olson, A. D. Thompson, K. D. Mandl, Integrating spatial epidemiology into a decision model for evaluation of facial palsy in children. Arch. Pediatr. Adolesc. Med. 165, 61–67 (2011). 21199982 10.1001/archpediatrics.2010.250 PMC3644029

[16] E. J. Nelson, A. I. Khan, A. M. Keita, B. J. Brintz, Y. Keita, D. Sanogo, M. T. Islam, Z. H. Khan, M. M. Rashid, D. Nasrin, M. H. Watt, S. M. Ahmed, B. Haaland, A. T. Pavia, A. C. Levine, D. L. Chao, K. L. Kotloff, F. Qadri, S. O. Sow, D. T. Leung, Improving antibiotic stewardship for diarrheal disease with probability-based electronic clinical decision support: A randomized crossover trial. JAMA Pediatr. 176, 973–979 (2022). 36036920 10.1001/jamapediatrics.2022.2535 PMC9425282

[17] M. Chan, M. A. Johansson, The incubation periods of dengue viruses. PLOS ONE 7, e50972 (2012). 23226436 10.1371/journal.pone.0050972 PMC3511440

[18] D. M. Watts, D. S. Burke, B. A. Harrison, R. E. Whitmire, A. Nisalak, Effect of temperature on the vector efficiency of Aedes aegypti for dengue 2 virus. Am. J. Trop. Med. Hyg. 36, 143–152 (1987). 3812879 10.4269/ajtmh.1987.36.143

[19] R. Barrera, M. Amador, A. J. MacKay, Population dynamics of Aedes aegypti and dengue as influenced by weather and human behavior in San Juan, Puerto Rico. PLOS Negl. Trop. Dis. 5, e1378 (2011). 22206021 10.1371/journal.pntd.0001378 PMC3243685

[20] G. S. Ribeiro, G. L. Hamer, M. Diallo, U. Kitron, A. I. Ko, S. C. Weaver, Influence of herd immunity in the cyclical nature of arboviruses. Curr. Opin. Virol. 40, 1–10 (2020). 32193135 10.1016/j.coviro.2020.02.004 PMC7434662

[21] V. Romeo-Aznar, L. Picinini Freitas, O. Gonçalves Cruz, A. A. King, M. Pascual, Fine-scale heterogeneity in population density predicts wave dynamics in dengue epidemics. Nat. Commun. 13, 996 (2022). 35194017 10.1038/s41467-022-28231-w PMC8864019

[22] J. Lourenço, M. Recker, Natural, persistent oscillations in a spatial multi-strain disease system with application to dengue. PLOS Comput. Biol. 9, e1003308 (2013). 24204241 10.1371/journal.pcbi.1003308 PMC3812071

[23] W. T. Lai, C. H. Chen, H. Hung, R. B. Chen, S. Shete, C. C. Wu, Recognizing spatial and temporal clustering patterns of dengue outbreaks in Taiwan. BMC Infect. Dis. 18, 256 (2018). 29866173 10.1186/s12879-018-3159-9 PMC5987425

[24] M. I. Estupiñán Cárdenas, V. M. Herrera, M. C. Miranda Montoya, A. Lozano Parra, Z. M. Zaraza Moncayo, J. P. Flórez García, I. Rodríguez Barraquer, L. Villar Centeno, Heterogeneity of dengue transmission in an endemic area of Colombia. PLOS Negl. Trop. Dis. 14, e0008122 (2020). 32925978 10.1371/journal.pntd.0008122 PMC7571714

[25] K. T. Thai, N. Nagelkerke, H. L. Phuong, T. T. Nga, P. T. Giao, L. Q. Hung, T. Q. Binh, N. V. Nam, P. J. De Vries, Geographical heterogeneity of dengue transmission in two villages in southern Vietnam. Epidemiol. Infect. 138, 585–591 (2010). 19653925 10.1017/S095026880999046X

[26] G. Ribeiro Dos Santos, B. Durovni, V. Saraceni, T. I. Souza Riback, S. B. Pinto, K. L. Anders, L. A. Moreira, H. Salje, Estimating the effect of the wMel release programme on the incidence of dengue and chikungunya in Rio de Janeiro, Brazil: A spatiotemporal modelling study. Lancet Infect. Dis. 22, 1587–1595 (2022). 36182679 10.1016/S1473-3099(22)00436-4 PMC9630156

[27] E. Fernández, M. Smieja, S. D. Walter, M. Loeb, A predictive model to differentiate dengue from other febrile illness. BMC Infect. Dis. 16, 694 (2016). 27876005 10.1186/s12879-016-2024-y PMC5120437

[28] C. Sa-Ngamuang, P. Haddawy, V. Luvira, W. Piyaphanee, S. Iamsirithaworn, S. Lawpoolsri, Accuracy of dengue clinical diagnosis with and without NS1 antigen rapid test: Comparison between human and Bayesian network model decision. PLOS Negl. Trop. Dis. 12, e0006573 (2018). 29912875 10.1371/journal.pntd.0006573 PMC6023245

[29] C. K. Bodinayake, L. G. Tillekeratne, A. Nagahawatte, V. Devasiri, W. Kodikara Arachchi, J. J. Strouse, O. M. Sessions, R. Kurukulasooriya, A. Uehara, S. Howe, X. M. Ong, S. Tan, A. Chow, P. Tummalapalli, A. D. De Silva, T. Østbye, C. W. Woods, D. J. Gubler, M. E. Reller, Evaluation of the WHO 2009 classification for diagnosis of acute dengue in a large cohort of adults and children in Sri Lanka during a dengue-1 epidemic. PLOS Negl. Trop. Dis. 12, e0006258 (2018). 29425194 10.1371/journal.pntd.0006258 PMC5823472

[30] R. P. Daumas, S. R. Passos, R. V. Oliveira, R. M. Nogueira, I. Georg, K. B. Marzochi, P. Brasil, Clinical and laboratory features that discriminate dengue from other febrile illnesses: A diagnostic accuracy study in Rio de Janeiro, Brazil. BMC Infect. Dis. 13, 77 (2013). 23394216 10.1186/1471-2334-13-77 PMC3574824

[31] A. C. Restrepo, P. Baker, A. C. Clements, National spatial and temporal patterns of notified dengue cases, Colombia 2007-2010. Trop. Med. Int. Health 19, 863–871 (2014). 24862214 10.1111/tmi.12325

[32] I. K. Yoon, A. Getis, J. Aldstadt, A. L. Rothman, D. Tannitisupawong, C. J. Koenraadt, T. Fansiri, J. W. Jones, A. C. Morrison, R. G. Jarman, A. Nisalak, M. P. Mammen Jr., S. Thammapalo, A. Srikiatkhachorn, S. Green, D. H. Libraty, R. V. Gibbons, T. Endy, C. Pimgate, T. W. Scott, Fine scale spatiotemporal clustering of dengue virus transmission in children and Aedes aegypti in rural Thai villages. PLOS Negl. Trop. Dis. 6, e1730 (2012). 22816001 10.1371/journal.pntd.0001730 PMC3398976

[33] P. Bhoomiboonchoo, R. V. Gibbons, A. Huang, I. K. Yoon, D. Buddhari, A. Nisalak, N. Chansatiporn, M. Thipayamongkolgul, S. Kalanarooj, T. Endy, A. L. Rothman, A. Srikiatkhachorn, S. Green, M. P. Mammen, D. A. Cummings, H. Salje, The spatial dynamics of dengue virus in Kamphaeng Phet, Thailand. PLOS Negl. Trop. Dis. 8, e3138 (2014). 25211127 10.1371/journal.pntd.0003138 PMC4161352

[34] R. Misslin, O. Telle, E. Daudé, A. Vaguet, R. E. Paul, Urban climate versus global climate change-what makes the difference for dengue? Ann. N. Y. Acad. Sci. 1382, 56–72 (2016). 27197685 10.1111/nyas.13084

[35] S. Flores Ruiz, S. Cabrera Romo, A. Castillo Vera, A. Dor, Effect of the rural and urban microclimate on mosquito richness and abundance in Yucatan State, Mexico. Vector Borne Zoonotic Dis. 22, 281–288 (2022). 35580213 10.1089/vbz.2021.0105 PMC9145259

[36] T. W. Scott, P. H. Amerasinghe, A. C. Morrison, L. H. Lorenz, G. G. Clark, D. Strickman, P. Kittayapong, J. D. Edman, Longitudinal studies of Aedes aegypti (Diptera: Culicidae) in Thailand and Puerto Rico: Blood feeding frequency. J. Med. Entomol. 37, 89–101 (2000). 15218911 10.1603/0022-2585-37.1.89

[37] N. Abdullah, N. C. Dom, S. A. Salleh, H. Salim, N. Precha, The association between dengue case and climate: A systematic review and meta-analysis. One Health 15, 100452 (2022). 36561711 10.1016/j.onehlt.2022.100452 PMC9767811

[38] S. Hossain, M. M. Islam, M. A. Hasan, P. B. Chowdhury, I. A. Easty, M. K. Tusar, M. B. Rashid, K. Bashar, Association of climate factors with dengue incidence in Bangladesh, Dhaka City: A count regression approach. Heliyon 9, e16053 (2023). 37215791 10.1016/j.heliyon.2023.e16053 PMC10192530

[39] C. A. Ouattara, T. I. Traore, S. Traore, I. Sangare, C. Z. Meda, L. G. B. Savadogo, Climate factors and dengue fever in Burkina Faso from 2017 to 2019. J. Public Health Afr. 13, 2145 (2022). 35720791 10.4081/jphia.2022.2145 PMC9202460

[40] N. Singh, R. K. Mall, T. Banerjee, A. Gupta, Association between climate and infectious diseases among children in Varanasi city, India: A prospective cohort study. Sci. Total Environ. 796, 148769 (2021). 34274660 10.1016/j.scitotenv.2021.148769

[41] H. C. Lu, F. Y. Lin, Y. H. Huang, Y. T. Kao, E. W. Loh, Role of air pollutants in dengue fever incidence: Evidence from two southern cities in Taiwan. Pathog. Glob. Health 117, 596–604 (2023). 36262027 10.1080/20477724.2022.2135711 PMC10617642

[42] M. A. F. Carneiro, B. Alves, F. S. Gehrke, J. N. Domingues, N. Sá, S. Paixão, J. Figueiredo, A. Ferreira, C. Almeida, A. Machi, E. Savóia, V. Nascimento, F. Fonseca, Environmental factors can influence dengue reported cases. Rev. Assoc. Med. Bras. 63, 957–961 (2017). 29451659 10.1590/1806-9282.63.11.957

[43] T. J. Betjeman, S. E. Soghoian, M. P. Foran, mHealth in Sub-Saharan Africa. Int. J. Telemed. Appl. 2013, 482324 (2013). 24369460 10.1155/2013/482324 PMC3867872

[44] D. J. Gubler, Dengue and dengue hemorrhagic fever. Clin. Microbiol. Rev. 11, 480–496 (1998). 9665979 10.1128/cmr.11.3.480 PMC88892

[45] H. Tissera, P. Samaraweera, M. de Boer, S. Gandhi, L. Malvaux, S. Mehta, P. Palihawadana, V. Vantomme, R. Paris, A. Schmidt, The burden of acute febrile illness attributable to dengue virus infection in Sri Lanka: A single-center 2-year prospective cohort study (2016-2019). Am. J. Trop. Med. Hyg. 106, 160–167 (2021). 34724624 10.4269/ajtmh.21-0604 PMC8733532

[46] S. Richardson, K. L. Dauber-Decker, T. McGinn, D. P. Barnaby, A. Cattamanchi, R. Pekmezaris, Barriers to the use of clinical decision support for the evaluation of pulmonary embolism: Qualitative interview study. JMIR Hum. Factors 8, e25046 (2021). 34346901 10.2196/25046 PMC8374661

[47] T. S. Ho, T. C. Weng, J. D. Wang, H. C. Han, H. C. Cheng, C. C. Yang, C. H. Yu, Y. J. Liu, C. H. Hu, C. Y. Huang, M. H. Chen, C. C. King, Y. J. Oyang, C. C. Liu, Comparing machine learning with case-control models to identify confirmed dengue cases. PLOS Negl. Trop. Dis. 14, e0008843 (2020). 33170848 10.1371/journal.pntd.0008843 PMC7654779

[48] A. S. Fathima, D. Manimeglai, Analysis of significant factors for dengue infection prognosis using the random forest classifier. Int. J. Adv. Comput. Sci. Appl. 6, 240–245 (2015).

[49] D. K. Ming, N. M. Tuan, B. Hernandez, S. Sangkaew, N. L. Vuong, H. Q. Chanh, N. V. Chau, C. P. Simmons, B. Wills, P. Georgiou, The diagnosis of dengue in patients presenting with acute febrile illness using supervised machine learning and impact of seasonality. Front. Digit. Health 4, 849641 (2022). 35360365 10.3389/fdgth.2022.849641 PMC8963938

[50] P. Kerdpanich, S. Kongkiatngam, D. Buddhari, S. Simasathien, C. Klungthong, P. Rodpradit, B. Thaisomboonsuk, T. Wongstitwilairoong, T. Hunsawong, K. B. Anderson, S. Fernandez, A. R. Jones, Comparative analyses of historical trends in confirmed dengue illnesses detected at public hospitals in Bangkok and Northern Thailand, 2002-2018. Am. J. Trop. Med. Hyg. 104, 1058–1066 (2020). 33319725 10.4269/ajtmh.20-0396 PMC7941814

[51] A. T. Huang, S. Takahashi, H. Salje, L. Wang, B. Garcia-Carreras, K. Anderson, T. Endy, S. Thomas, A. L. Rothman, C. Klungthong, A. R. Jones, S. Fernandez, S. Iamsirithaworn, P. Doung-Ngern, I. Rodriguez-Barraquer, D. A. T. Cummings, Assessing the role of multiple mechanisms increasing the age of dengue cases in Thailand. Proc. Natl. Acad. Sci. U.S.A. 119, e2115790119 (2022). 35533273 10.1073/pnas.2115790119 PMC9171776

[52] K. B. Anderson, D. Buddhari, A. Srikiatkhachorn, G. D. Gromowski, S. Iamsirithaworn, A. L. Weg, D. W. Ellison, L. Macareo, D. A. T. Cummings, I.-K. Yoon, A. Nisalak, A. Ponlawat, S. J. Thomas, S. Fernandez, R. G. Jarman, A. L. Rothman, T. P. Endy, An Innovative, prospective, hybrid cohort-cluster study design to characterize dengue virus transmission in multigenerational Households in Kamphaeng Phet, Thailand. Am. J. Epidemiol. 189, 648–659 (2020). 31971570 10.1093/aje/kwaa008 PMC7393304

[53] G. Ribeiro Dos Santos, D. Buddhari, S. Iamsirithaworn, D. Khampaen, A. Ponlawat, T. Fansiri, A. Farmer, S. Fernandez, S. Thomas, I. Rodriguez Barraquer, A. Srikiatkhachorn, A. T. Huang, D. A. T. Cummings, T. Endy, A. L. Rothman, H. A.-O. Salje, K. A.-O. Anderson, Individual, household, and community drivers of dengue virus infection risk in Kamphaeng Phet Province, Thailand. J. Infect. Dis. 226, 1348–1356 (2022). 35512137 10.1093/infdis/jiac177 PMC9574660

[54] A. Sarica, A. Cerasa, A. Quattrone, Random forest algorithm for the classification of neuroimaging data in alzheimer's disease: A systematic review. Front. Aging Neurosci. 9, 329 (2017). 29056906 10.3389/fnagi.2017.00329 PMC5635046

[55] S. Y. Peng, Y. C. Chuang, T. W. Kang, K. H. Tseng, Random forest can predict 30-day mortality of spontaneous intracerebral hemorrhage with remarkable discrimination. Eur. J. Neurol. 17, 945–950 (2010). 20136650 10.1111/j.1468-1331.2010.02955.x

